# Technological Advances of Cryopreservation in Ovarian Tissue for Female Children: Exploring the Molecular Insights and Mechanisms

**DOI:** 10.3390/ijms27125186

**Published:** 2026-06-08

**Authors:** Hsin-Hung Wu

**Affiliations:** 1 Reproductive Medicine Center, Department of Obstetrics and Gynecology, Changhua Christian Hospital, 135 Nanhsiao Street, Changhua 50006, Taiwan; 58436@cch.org.tw or wuwang97@gmail.com; Tel.: +886-4-7238595 (ext. 4622); 2 Department of Post-Baccalaureate Medicine, College of Medicine, National Chung Hsing University, Taichung 40227, Taiwan; 3 Institute of Medical Research, College of Health Sciences, Chang Jung Christian University, Tainan 711301, Taiwan

**Keywords:** ovarian tissue cryopreservation, fertility preservation, prepubertal girls, molecular mechanisms, follicle burnout, ischemia–reperfusion injury, tissue engineering, oncofertility

## Abstract

Ovarian tissue cryopreservation (OTC) has emerged as the only viable fertility preservation strategy for prepubertal girls and adolescent cancer patients facing gonadotoxic treatments. While OTC has transitioned from an experimental procedure to an established clinical practice, the functional longevity of transplanted grafts remains limited by massive follicle depletion. This review synthesizes recent technological advances in OTC for female children, with a particular focus on the underlying molecular mechanisms and innovative protective strategies. We systematically evaluate pre-cryopreservation assessments, surgical harvesting techniques such as medulla-sparing biopsies, and the comparative efficacy of slow freezing versus vitrification in preserving stromal and follicular integrity. Central to this discussion are the molecular drivers of post-transplantation injury, including ischemia–reperfusion-induced oxidative stress and the iatrogenic over-activation of the PI3K/Akt/mTOR signaling pathway, which leads to follicular “burnout.” Furthermore, we explore targeted pharmacological interventions, such as the dual-drug application of VEGFA and rapamycin, alongside emerging bioengineering frontiers including decellularized extracellular matrix scaffolds and 3D-printed bioprosthetic ovaries. Clinical outcomes are also summarized, highlighting high rates of endocrine recovery (~95%) and promising live birth rates (~28%), predominantly through natural conception. By integrating deep molecular insights with advanced tissue engineering, this review provides a comprehensive framework for optimizing long-term fertility restoration and improving the quality of survivorship for young female cancer survivors.

## 1. Introduction

Recent epidemiological data highlight a rising global cancer incidence among adolescents and young adults (AYAs) aged 15 to 39, with females exhibiting an age-standardized incidence rate of 52.9 per 100,000 [1]. However, advances in modern oncological therapies have significantly improved the overall 5-year relative survival rate of AYA cancer patients to approximately 83–87% [2]. Consequently, addressing future fertility needs has emerged as a critical component in the comprehensive survivorship care of this demographic. Given that antineoplastic treatments, particularly chemotherapy and radiotherapy, are highly gonadotoxic and frequently precipitate premature ovarian insufficiency (POI), timely fertility preservation counseling and intervention prior to therapy are imperative [3].

Current established fertility preservation modalities for females encompass embryo and mature oocyte cryopreservation, alongside ovarian tissue cryopreservation (OTC) [4]. Notably, OTC circumvents the need for time-consuming hormonal stimulation, thereby preventing critical delays in oncological management. Crucially, it remains the sole viable fertility preservation strategy for prepubertal girls. In recent years, OTC has successfully transitioned from an experimental procedure to a standardized clinical practice endorsed by international guidelines [5,6,7]. As of 2017, over 130 live births have been documented globally, with subsequent systematic reviews confirming a continued exponential growth [4,8].

Despite its profound clinical potential, the widespread application of OTC encounters significant physiological challenges. Post-transplantation ischemia–reperfusion injury and oxidative stress induce massive follicular loss, drastically restricting the functional lifespan of the graft to an average of four to five years [9]. Furthermore, there is an inherent risk of reintroducing malignant cells, which is particularly concerning for patients with hematological malignancies. To navigate these clinical bottlenecks, future research must prioritize the development of safer tissue utilization strategies and interventions to mitigate post-transplantation follicle depletion. This review aims to synthesize current technological advancements in OTC for female children, comprehensively exploring existing challenges and future optimization strategies from the perspective of underlying molecular mechanisms and targeted protective interventions.

## 2. Pre-Cryopreservation Status: Ovarian Function Evaluation and Chemotherapy Impact Results

### 2.1. Evaluation of Ovarian Function Before Cryopreservation

Prior to initiating OTC, an accurate assessment of baseline ovarian function and the follicular reserve is fundamental for establishing optimal fertility preservation strategies. Among various predictive parameters, chronological age serves as the most critical determinant of OTC success. Histological analyses reveal a significant, linear negative correlation between primordial follicle density in the ovarian cortex and age. In prepubertal girls (under 10 years of age), the average follicle density can reach 20.36 ± 19.03 follicles/mm^2^. This density sharply decreases to 4.13 ± 2.9 follicles/mm^2^ between the ages of 10 and 15, and further declines to 1.63 ± 3.35 follicles/mm^2^ in individuals over 15 years old [10]. However, the results showed that follicles in the human ovarian cortex were unevenly distributed, resulting in a huge variation in follicular density as prepared for cryopreservation. These findings highlight that removal of a whole ovary may benefit the woman more than the removal of a small biopsy for fertility preservation [10].

Conversely, anti-Müllerian hormone (AMH) levels demonstrate limited prognostic value for determining actual primordial follicle density in younger populations. Studies indicate that in females under 20 years old, AMH concentrations exhibit no significant correlation with follicular density [11]. This discrepancy arises because, although the follicle pool is highly dense during childhood, AMH secretion does not reach its physiological peak until early adulthood (ages 21–25) [12]. Before puberty, AMH levels are naturally lower and more variable because the pool of growing preantral and small antral follicles (which actively secrete AMH) is relatively dormant compared to adult women. Therefore, we clarified that while a low AMH level in a young child should be interpreted with extreme caution and contextualized with chronological age and prior gonadotoxic exposure, it cannot serve as a standalone exclusionary criterion for OTC.

### 2.2. Feasibility of OTC Following Prior Chemotherapy Exposure

Due to the aggressive nature of certain malignancies, some adolescent patients are compelled to initiate chemotherapy before OTC can be performed. While traditional paradigms suggest that chemotherapy profoundly damages ovarian tissues, recent retrospective studies indicate that prior exposure to chemotherapy does not significantly impair the functional recovery of cryopreserved ovarian grafts [13]. Recent evidence suggests that prior exposure to chemotherapy should not be considered an absolute contraindication for ovarian tissue cryopreservation (OTC). Although traditional perspectives assumed chemotherapy would severely damage ovarian tissues, clinical data show robust outcomes for exposed cohorts, including a 93% endocrine recovery rate and a 32% live birth rate [13]. These success rates counterintuitively surpassed those of unexposed cohorts, primarily due to differences in the underlying pathologies of the patients. Notably, low-dose alkylating agents do not cause significant adverse effects on overall follicle density, cellular viability, or tissue morphology [14]. However, higher doses and bifunctional alkylating agents are associated with more concerning in vitro and long-term effects, such as increased densities of atretic follicles, lower long-term graft survival, elevated apoptosis rates, and significantly reduced vascularization [15]. Ultimately, while OTC remains a highly feasible option following partial or low-dose chemotherapy, harvesting ovarian tissues prior to the initiation of any gonadotoxic treatments remains the ideal clinical scenario to maximize follicle quality and graft longevity [15,16].

### 2.3. Molecular Mechanisms of Chemotherapy-Induced Oocyte Damage

**Figure 1:**
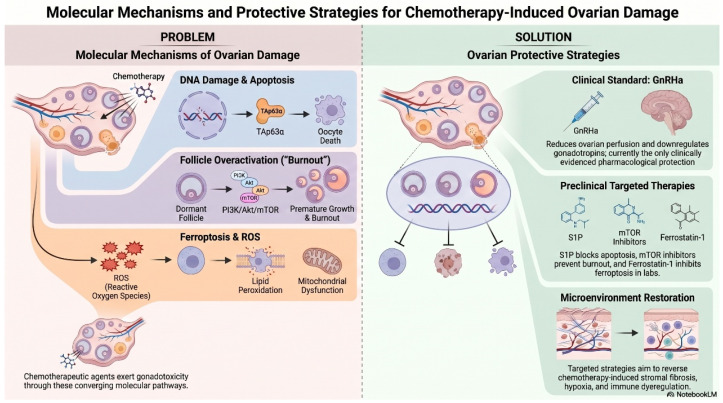
Molecular mechanisms of chemotherapy-induced ovarian damage and corresponding protective strategies. The figure illustrates the primary pathways of gonadotoxicity and potential therapeutic interventions. (**Left**) Pathogenesis of ovarian damage: Chemotherapeutic agents exert gonadotoxicity through three converging molecular pathways: (1) DNA damage and apoptosis, leading to TAp63α-mediated oocyte death; (2) Follicle overactivation (“burnout”), wherein dormant follicles are forced into premature growth via the PI3K/Akt/mTOR signaling pathway; and (3) Ferroptosis and oxidative stress, characterized by the accumulation of reactive oxygen species (ROS), subsequent lipid peroxidation, and mitochondrial dysfunction. (**Right**) Ovarian protective strategies: Current and emerging interventions include: (1) Clinical standard (GnRHa), which reduces ovarian perfusion and downregulates gonadotropins; (2) Preclinical targeted therapies, utilizing S1P to block apoptosis, mTOR inhibitors to prevent follicle burnout, and Ferrostatin-1 to inhibit ferroptosis; and (3) Microenvironment restoration, with targeted strategies aiming to reverse chemotherapy-induced stromal fibrosis, hypoxia, and immune dysregulation. (Data generated using NotebookLM).

The toxicity of chemotherapeutic agents on the ovarian reserve is a multifaceted process. Recent research has shifted the paradigm from viewing oocyte death as a single physiological event to understanding it as a complex interplay of direct DNA damage, systemic metabolic failures (ferroptosis), and profound structural degradation of the surrounding tissue. From a molecular perspective, the gonadotoxicity exerted by chemotherapeutic agents depletes the ovarian reserve through multiple converging pathological pathways (Figure 1).

#### 2.3.1. DNA Damage and the Apoptotic Cascade

Apoptosis is now widely considered the primary and most immediate mechanism driving the loss of primordial follicles in humans following chemotherapy exposure.

##### The TAp63α Master Switch

The protein TAp63α is the absolute central regulator of oocyte death [17]. In a healthy oocyte, it remains dormant. Upon detecting severe DNA double-strand breaks, a highly specific sequential kinase cascade is initiated: sensors (ATM/ATR) signal to intermediary kinases (CHK1/CHK2), which ultimately activate TAp63α [18].

##### Detailed Kinase Interactions

CHK2 acts as the “priming” kinase, while casein kinase 1 (CK1) serves as the “executioner” kinase. These kinases work in a semiredundant manner; if both the CHK1 and CHK2 pathways simultaneously transmit distress signals to the TRP53/TAp63α network, almost all affected oocytes are completely eliminated [19].

##### Agent-Specific Molecular Triggers (Cisplatin)

A 2024 study revealed that cisplatin triggers apoptosis in a highly specific way—it induces the de novo synthesis of new TAp63α proteins rather than just tetramerizing existing ones. CK1-mediated phosphorylation is absolutely essential to activate this newly synthesized protein. Furthermore, cisplatin primarily utilizes the ATR → CHK1 → TAp63α pathway, which operates independently of hyperphosphorylation [18,19].

##### Agent-Specific Molecular Triggers (Alkylating Agents and Radiation)

Alkylating agents (like cyclophosphamide) and topoisomerase II poisons are the most potent activators due to the massive, direct DNA damage they inflict [20]. Radiation, conversely, relies on the ATM → CHK2 → TAp63α pathway, which specifically requires hyperphosphorylation to trigger cell death [21].

##### The Execution Phase

Once TAp63α is fully active, it upregulates the expression of PUMA and NOXA, which act as the final pro-apoptotic effectors that dismantle the primordial follicle [22].

#### 2.3.2. The Controversy of Follicle Overactivation (“Burnout”)

For years, the “burnout” theory suggested chemotherapy destroyed the ovarian reserve by forcing dormant primordial follicles to wake up and grow rapidly, prematurely exhausting the supply. This hypothesis is now highly disputed in human models.

##### Refuting the Burnout Theory

A landmark 2021 study utilizing single-cell RNA sequencing on human primordial follicles found absolutely no evidence that the PI3K/PTEN/Akt pathway (the primary driver of follicle activation) was activated after cyclophosphamide exposure [23]. Instead, the expression of pro-Akt genes drastically dropped, while DNA damage and apoptosis markers spiked. A subsequent 2024 study in prepubertal mice confirmed this lack of activation at both the transcriptional and histological levels, noting that follicles simply died via apoptosis within 24 h [24].

##### Nuances and Counter-Evidence

Despite the shift away from the burnout theory, some pathway dysregulation still occurs. In certain models, less than one-third of follicle loss can be purely attributed to immediate apoptosis, leaving room for secondary mechanisms [25].

##### Targeted Protections

Suppressing these activation pathways still yields protective results. For example, using temsirolimus (an mTOR inhibitor) alongside recombinant AMH completely prevented cyclophosphamide-induced loss in mice [26]. Furthermore, a 2026 study demonstrated that treatments combining retinoic acid and calcitriol protect follicles by forcing the reversal of PI3K/Akt activity and preventing FOXO3a nuclear export, which actively suppresses oocyte transcription and limits DNA damage [27].

#### 2.3.3. Ferroptosis: The Emerging Metabolic Threat

Since 2023, researchers have recognized that cell death is not just driven by nuclear DNA damage; chemotherapy fundamentally destroys the cell’s metabolic and lipid balance through a process called ferroptosis.

##### The Biochemical Failure

Chemotherapeutic agents (specifically cisplatin, cyclophosphamide, and doxorubicin) induce massive iron overload, severe lipid peroxidation, and mitochondrial dysfunction inside ovarian granulosa cells. The drugs cause excessive reactive oxygen species (ROS) production and mitochondrial superoxide accumulation [28].

##### Core Molecular Dysregulation

This is driven by the collapse of the SLC7A11-GSH-GPX4 protective axis [29]. Specifically, GPX4 (which normally protects against lipid peroxidation) is severely downregulated, while ACSL4 is upregulated [30]. The HO-1 (heme oxygenase-1) pathway accelerates the damage by promoting further iron release and ROS generation [31].

##### The Mitophagy Vicious Cycle

A cutting-edge 2026 study discovered a compounding effect: cyclophosphamide disrupts iron homeostasis, causing Fe^2+^ to pool inside mitochondria [32]. This triggers aggressive mitophagy (the cell eating its own mitochondria), which ironically exacerbates the mitochondrial damage and sensitizes the cell further to ferroptosis.

##### Therapeutic Rescues

Preclinical trials have successfully mitigated this damage using specific ferroptosis inhibitors like ferrostatin-1 and targeted iron chelators like deferoxamine [33]. Additionally, estradiol has proven highly effective at blocking ferroptosis by activating the protective ESR2/Sirt1/Nrf2 signaling pathway [34].

#### 2.3.4. Destruction of the Ovarian Microenvironment

Even if an oocyte survives the direct chemical attack, chemotherapy leaves behind a hostile “soil”—a ruined ovarian stroma that prevents future follicle development.

##### Structural and Vascular Collapse

The physical structure of the ovary is severely altered, featuring extracellular matrix (ECM) deposition, severe stromal fibrosis, blood vessel damage, and cellular senescence. A classic 2007 study first noted this cortical fibrosis and vascular destruction in human ovaries, observing focal areas where follicles had completely vanished [35].

##### Proteomic Alterations

A 2025 proteomic analysis of human ovarian tissue showed that first-line chemotherapy fundamentally alters cellular programming. It massively upregulates pathways linked to destructive immune responses (such as complement C3), extreme hypoxia (SELENBP1), and apoptosis (KRT18). Simultaneously, essential cell cycle and DNA repair pathways are shut down [36].

##### Fibrotic Pathways and Rescues

Mechanistically, chemotherapy forces stromal cells to activate the TGF-β1/Smad2/3 pathway, which transforms healthy cells into activated fibroblasts that dump excess collagen, causing fibrosis [37].

##### Stem Cell Therapy

A 2026 study proved that introducing bone marrow-derived mesenchymal stem cells (BMSCs) can rescue the ovary by upregulating RGS3 (regulator of G protein signaling 3). RGS3 actively suppresses the TGF-β1/Smad2/3 pathway and boosts VEGF, thereby dissolving fibrosis and repairing blood vessels [38].

##### Metformin

The common diabetes drug metformin has also shown promise in 2025 trials. It targets the MIF/CD74 axis to reprogram the harmful communication between macrophages and fibroblasts, actively alleviating cyclophosphamide-induced fibrosis [39].

### 2.4. Potential Ovarian Protective Strategies During Chemotherapy

To counteract these molecular mechanisms of damage, various pharmacological protective strategies have been explored. In current clinical practice, gonadotropin-releasing hormone agonists (GnRHa) are the only agents with established clinical evidence for ovarian protection, acting to downregulate gonadotropin secretion and reduce ovarian perfusion [40,41]. However, guidelines emphasize that GnRHa should serve as an adjuvant therapy and must not replace established methods like OTC [5,6,7]. In preclinical stages, novel targeted therapeutics, such as Sphingosine-1-phosphate (S1P) for blocking apoptotic pathways, mTOR inhibitors for preventing follicle “burnout,” and Ferrostatin-1 for inhibiting ferroptosis, show immense promise, though clinical translation remains hindered by potential interference with antineoplastic efficacy [25,42,43].

## 3. Ovarian Tissue Harvest

### 3.1. Laparoscopic Ovarian Tissue Retrieval Methods

Ovarian tissue cryopreservation is typically performed utilizing standard three-port laparoscopy. In clinical practice, surgeons generally employ either complete oophorectomy (utilized in ~96% of cases) or an ovarian cortical biopsy featuring a medulla-sparing technique. In a UK national survey of pediatric centers, 96% of respondents performed total oophorectomy [44], though practice varies by patient age and center, with partial cortical excision more common in adult patients. The medulla-sparing approach, which involves solely dissecting the cortical tissue while preserving the medullary vascular supply, is highly advantageous as it maximizes the retrieval of primordial follicles while minimizing collateral tissue injury [45]. To mitigate thermal damage to the fragile ovarian cortex, cold scissors are preferred over electrocautery, and the retrieved tissue is extracted via a retrieval bag [44].

### 3.2. Impact of Time from Retrieval to Cryopreservation

The duration between surgical extraction and the initiation of cryopreservation is a critical determinant of follicle viability. Clinical evidence indicates that transporting ovarian tissue on ice for up to 6 h yields robust follicle survival rates (averaging 84%), with no significant difference compared to tissues processed immediately. Extending transport time up to 21 h does not significantly compromise the structural integrity of primordial follicles; however, follicle apoptosis increases significantly once this period exceeds 20.58 h [46]. Importantly, once cellular metabolic activity is completely halted and the tissue is stably maintained below (typically at in liquid nitrogen), the duration of storage, even exceeding 10 to 20 years, does not significantly exacerbate cellular damage or compromise primordial follicle viability compared to short-term storage [47]. The warming phase is just as critical as the freezing phase in determining tissue viability. Particularly for vitrification, ultra-rapid warming is essential to cross the dangerous temperature zones quickly, thereby preventing the recrystallization of intracellular ice (devitrification) and mitigating osmotic shock, which are primary drivers of mechanical and membrane damage during the recovery phase.

## 4. Ovarian Tissue Cryopreservation (OTC)

### 4.1. Methodological Approaches: Slow Freezing and Vitrification

Ovarian tissue cryopreservation (OTC) primarily relies on two methodologies, slow freezing and vitrification, that yield comparable clinical outcomes, demonstrating no significant differences in follicular viability, morphology, or primordial follicle density [48,49]. Slow freezing is the established clinical standard responsible for the majority of live births worldwide, typically utilizing 1.5 M DMSO and 0.1 M sucrose with programmed cooling to −140 °C [49,50,51]. In contrast, vitrification employs ultra-rapid cooling and high concentrations of cryoprotectants (such as DMSO and ethylene glycol) to completely bypass ice crystal formation [49,50]. While vitrification offers potential benefits such as reduced DNA damage, better stromal cell preservation, increased efficiency, and lower costs, it currently lacks the extensive clinical data of slow freezing and presents safety considerations regarding exposure to high cryoprotectant concentrations and direct liquid nitrogen contact [50]. Ultimately, the choice between the two methods depends on institutional experience, available equipment, and the need for further long-term clinical data to fully establish vitrification’s efficacy [52,53].

### 4.2. Molecular and Cellular Mechanisms of Cryopreservation-Induced Damage

The transition of complex, heterogeneous ovarian tissue to ultra-low temperatures (below −150 °C) triggers a cascade of cellular stress. The cryopreservation process inevitably inflicts injury, primarily manifesting as altered nuclear architecture, DNA strand breaks, significant oxidative stress, and apoptosis. At the molecular and transcriptomic levels, this damage occurs through several interconnected pathways [54].

#### 4.2.1. Physical Disruption and Osmotic Shock

Traditional cryopreservation relies on cryoprotective agents (CPAs) to displace intracellular water. If dehydration is inadequate, expanding intracellular ice crystals physically puncture cell membranes and organelles, leading to the observed altered nuclear architecture and fatal DNA strand breaks post-thaw [54].

#### 4.2.2. Ischemia/Hypoxia and Oxidative Stress

The freeze–thaw cycle subjects’ cells to severe ischemia and hypoxia, triggering mitochondrial dysfunction and the massive overproduction of reactive oxygen species (ROS) [52]. This significant oxidative stress induces lipid peroxidation, damages structural proteins, and ultimately initiates apoptotic cascades [55].

### 4.3. Heterogeneity of the Ovarian Microenvironment and Cell-Specific Cryosensitivity

Single-cell spatial transcriptomic analyses reveal the highly heterogeneous nature of ovarian tissue, indicating that stromal cells and perivascular cells exhibit significantly higher cryosensitivity compared to follicular cells. The underlying mechanisms and characteristics are as follows:

#### 4.3.1. Differential Activation of Stress Response Pathways

Cryopreservation significantly alters pathways related to focal adhesion, oxidative stress, and apoptosis, which is particularly evident in stromal and perivascular cells. Following vitrification and rapid warming, the FOS/AP-1 stress pathway is markedly activated in perivascular and granulosa cells, suggesting an exacerbation of metabolic impairment. Post-thaw, the number of FOS-positive perivascular cells notably increases, whereas PTGDS-positive stromal cells decrease [56,57].

#### 4.3.2. Transcriptomic Alterations and Apoptotic Pathways

Oocytes: Cryopreservation primarily disrupts pathways involved in the cell cycle and meiosis, though such damage is typically not irreversible [56].

Stromal Cells: Alterations occur in the expression of WNT signaling and hormonal regulation pathway genes. Slow freezing leads to a significant decrease in the expression of the cell cycle gene CCND2 [10], whereas vitrification induces the overexpression of steroidogenic genes (e.g., CYP11A) and increases the BAX:BCL-2 ratio, indicating greater apoptotic stress within the stromal compartment [58].

#### 4.3.3. Spatial Distribution of Mitochondrial Dysfunction

Both slow freezing and vitrification lead to reduced mitochondrial activity and decreased intracellular ROS levels. Mitochondrial dysfunction progresses from the outer to the inner sections of the ovarian cortex; vitrified samples demonstrate a significantly more severe reduction in mitochondrial activity in the inner sections [59].

#### 4.3.4. Structural Vulnerability and Limited Self-Repair Capacity

Structural Damage: Slow freezing often causes interstitial edema, stromal cell vacuolization, and chromatin clumping. Vitrification produces moderately dispersed chromatin and homogeneous cytoplasm accompanied by slight vacuolization [50].

Repair Capacity: While primordial follicles exhibit promising self-repair potential, the recovery capacity of stromal cells is highly limited. After 5 days of tissue culture, slow freezing maintains the DNA integrity of stromal cells more effectively than vitrification; however, immediately post-thaw, vitrification better preserves the DNA integrity of primordial follicles [60].

#### 4.3.5. Physical Toxicity of Cryoprotective Agents (CPAs)

The high concentrations of CPAs required for vitrification can induce DMSO toxicity. When DMSO concentrations exceed optimal levels, it causes mitochondrial degeneration, abnormal chromatin condensation, cell vacuolization, and extracellular matrix swelling in both stromal and follicular cells [61].

### 4.4. Cryopreservation-Induced Damage and Clinical Outcomes (Figure 2)

The cryopreservation process inevitably inflicts injury, primarily manifesting as altered nuclear architecture, DNA strand breaks, significant oxidative stress, and apoptosis. Recent transcriptomic analyses identify stromal and perivascular cells as the most cryosensitive populations [57,62]. While systematic reviews indicate no significant difference in the proportion of intact primordial follicles preserved between the two methods [10,63], vitrification results in significantly less DNA damage and demonstrates marked superiority in preserving stromal cell integrity [50,62,64]. Regarding pregnancy outcomes, the meta-analysis showed that slow freezing achieves a cumulative pregnancy rate of 37%, compared to 44% for vitrification [65].

**Figure 2:**
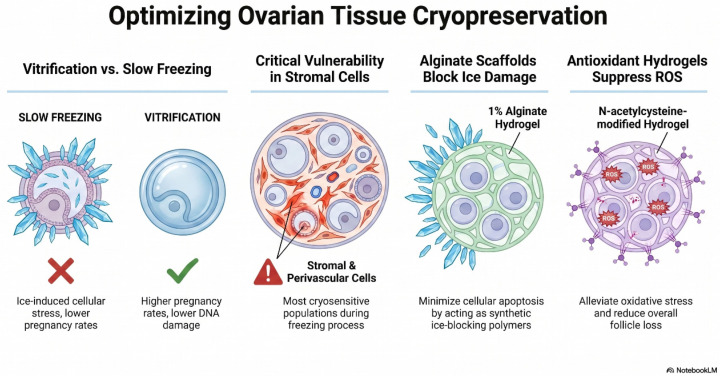
Overview of Strategies for Optimizing Ovarian Tissue Cryopreservation. This figure illustrates current approaches and novel strategies aimed at improving ovarian tissue cryopreservation (OTC) outcomes. Comparison of Slow Freezing vs. Vitrification: The effectiveness of two fundamental freezing techniques is compared. Slow Freezing (**left**) is characterized by the formation of ice crystals (red X indicates detrimental effect), leading to ice-induced cellular stress and, ultimately, lower pregnancy rates. Conversely, Vitrification (**right**, indicated by green checkmark) employs rapid cooling to bypass ice formation, resulting in higher pregnancy rates and lower levels of DNA damage. Identification of Critical Vulnerability in Stromal Cells: A cross-sectional view of the ovarian tissue reveals the inherent cell types. While follicles are the therapeutic target, the Stromal and Perivascular Cells (highlighted by the red warning triangle) are identified as the most cryosensitive populations during the conventional freezing process, representing a major limiting factor for success. Ice-Blocking Hydrogel Scaffolds: A novel approach utilizes a synthetic, non-cytotoxic, 1% Alginate Hydrogel scaffold (depicted in green) to block ice damage. The hydrogel encapsulates the ovarian tissue, minimizing ice crystal formation within the tissue structure and thereby reducing cellular apoptosis. Antioxidant-Modified Hydrogels to Suppress ROS: To further improve survival, an N-acetylcysteine (NAC)-modified Hydrogel (depicted in purple) is employed. This antioxidant-enhanced hydrogel actively suppresses Reactive Oxygen Species (ROS) generation within the tissue, alleviating oxidative stress and significantly reducing overall follicle loss (Data generated using NotebookLM).

### 4.5. Novel Biomaterials and Cryoprotectants

To mitigate cryoinjury, contemporary research leverages novel biomaterials. The application of synthetic ice-blocking polymers and biological scaffolds (e.g., 1% alginate hydrogels) during the freezing process significantly reduces the expression of apoptosis-related genes and diminishes local oxidative stress [66]. Furthermore, antioxidant interventions, such as N-acetylcysteine-modified hydrogels loaded with mesenchymal stem cell-conditioned medium, profoundly suppress ROS generation and reduce follicle loss [67].

## 5. Ovarian Tissue Transplantation (OTT)

### 5.1. Preoperative Optimization and Site Selection

The pharmacological preparation of the recipient’s vascular bed prior to surgery (e.g., perioperative administration of transdermal estrogen and low-dose aspirin) facilitates vascularization and improves transplantation outcomes [68]. Regarding anatomical placement, clinical data strongly advocate for the orthotopic approach (transplantation to the remaining ovary or pelvic peritoneum), which achieves a live birth rate of 44%, compared to only 5% for heterotopic transplantation [69].

### 5.2. Molecular Pathology and Mechanisms of Injury in Ovarian Tissue Transplantation (OTT)

Ovarian Tissue Transplantation (OTT) is a vital fertility preservation strategy, but its clinical efficacy is severely hampered by massive follicular loss post-grafting. The destruction of the ovarian reserve is not a single event, but rather a cascading, bipartite pathological mechanism: a physical assault from ischemia–reperfusion injury, followed immediately by a biochemical rebellion known as follicular burnout.

#### 5.2.1. The Hypoxic Chokehold and the Reperfusion Paradox

The immediate post-transplantation period forces the ovarian graft to survive in a hostile, avascular environment.

##### The Prolonged Ischemic Window

Current in vivo studies reveal that the grafted tissue endures severe, unrelenting hypoxia for approximately 7 days before any meaningful revascularization is established [70].

##### Metabolic Starvation via Microdialysis

The severity of this starvation has been mapped using in vivo microdialysis in human xenograft models. This technique demonstrated that the tissue is forced into a prolonged state of anaerobic metabolism. Specifically, localized lactate levels remain alarmingly higher than glucose levels until day 10 post-transplantation, physically proving the extended duration of ischemic stress [71].

##### The Dual-Peak ROS Assault

When blood flow finally returns (reperfusion), it paradoxically causes massive damage. The reintroduction of oxygen triggers massive reactive oxygen species (ROS) generation, which does not occur as a single spike, but rather in two distinct, destructive peaks around day 10 and 17 post-transplantation [71,72].

##### Lipid Peroxidation and Mitochondrial Collapse

This oxidative shock physically destroys cell structures. It drives up levels of malondialdehyde (MDA), a primary biomarker of severe lipid peroxidation and membrane damage [73]. Furthermore, high-resolution respirometry reveals catastrophic mitochondrial dysfunction, marked by a dramatic decrease in oxygen consumption capacity across all cellular respiratory states [74]. Astonishingly, the metabolic shock of transplantation inflicts more intense damage on mitochondrial activity than the extreme thermal stress of cryopreservation itself. Ultimately, this oxidative siege forces follicles into widespread apoptosis and necrosis [60].

#### 5.2.2. Mechanotransduction and “Follicular Burnout”

While ischemia kills follicles directly, a second, more insidious mechanism depletes the surviving reserve. The combined trauma of freezing, thawing, and surgical grafting triggers a severe dysregulation of the intra-ovarian signaling pathways that dictate whether a follicle sleeps or grows.

##### Hyperactivation of the PI3K/Akt/mTOR Axis

Under normal biological conditions, the PI3K/PTEN/Akt and mTOR pathways act as strict gatekeepers, enforcing the dormancy of primordial follicles [75]. However, molecular analyses (including Western blots) confirm that the stress of OTT aggressively upregulates and activates both the Akt and mTOR pathways compared to fresh, unmanipulated ovarian tissue [76].

##### Massive Spontaneous Activation

Stripped of their regulatory brakes, approximately 80% of the entire primordial follicle pool spontaneously and erroneously activates during the early post-transplant period. Follicle activation occurs within 3 days of transplantation. Xenograft studies demonstrate a significant decrease in primordial follicles and a corresponding increase in growing follicles by day 3 post-grafting (*p* = 0.01) [77]. More than 50% of follicles become atretic after 3 days, with an additional 50% after 7 days of grafting [77].

##### Hippo Pathway Disruption via Fragmentation

The physical act of preparing the graft requires tissue fragmentation, which severs cellular connections and alters mechanotransduction. This physical trauma directly disrupts the Hippo signaling pathway [10]. Consequently, YAP (Yes-associated protein) undergoes nuclear translocation within the granulosa cells, sending false growth signals that further accelerate premature follicle recruitment [78,79]. However, there is conflicting evidence of fragmentation-induced activation in human ovarian tissues. The study findings indicate that fragmentation may be ineffective at activating follicle growth in human ovarian cortex through the Hippo pathway mechanism [80].

##### The “Burnout” Phenomenon

This uncontrolled, massive awakening of the resting follicle pool leads to rapid depletion—a clinical phenomenon termed “burnout.” Burnout drastically reduces the active lifespan of the ovarian graft, rendering it functional for a much shorter time than biologically intended.

### 5.3. Protective Strategies and Therapeutic Frontiers in Ovarian Tissue Transplantation (OTT) (Figure 3)

To counteract the destruction of the ovarian reserve, current protective strategies involve antioxidant therapies, pro-angiogenic interventions (e.g., VEGFA), and mTOR pathway inhibition (e.g., Rapamycin). A promising dual-drug strategy combining VEGFA and Rapamycin simultaneously drives revascularization while suppressing follicle over-activation [81,82]. Antioxidant therapies, including glutathione (GSH) and ulinastatin (UTI), effectively reduce oxidative stress and inflammation while increasing VEGF and CD31 expression [83]. N-acetylcysteine (NAC) demonstrates antioxidant, anti-inflammatory, and anti-apoptotic properties in xenotransplanted human ovarian tissue, and protects ovarian follicles from ischemia–reperfusion injury [84]. NAC coupled with hydrogel carriers significantly inhibits ROS generation [69]. Anti-apoptotic drugs like the pan-caspase inhibitor Z-VAD-FMK and Sphingosine-1-phosphate (S1P) maintain follicle density [85]. When S1P is co-administered with Follicle-Stimulating Hormone (FSH), it actively preserves the primordial pool via the AKT/mTOR pathway [86]. Hormonal supplementation using Recombinant Anti-Müllerian Hormone (AMH) attenuates loss by regulating Tsc1, p-s6k, and Gdf-9 [87].

**Figure 3:**
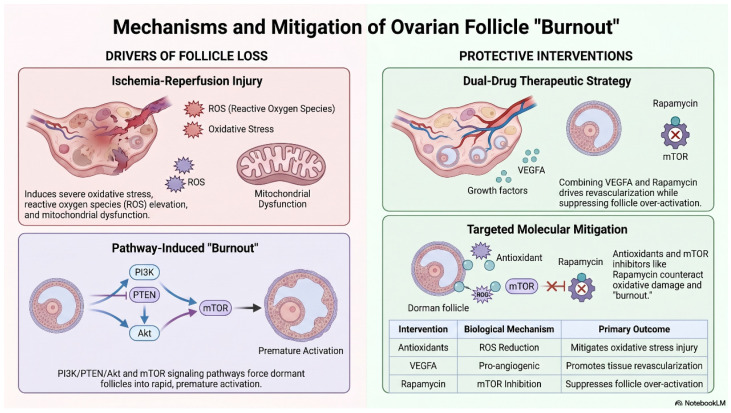
Mechanisms and Mitigation Strategies of Ovarian Follicle “Burnout.” (**Left**) Drivers of Follicle Loss: The figure illustrates two primary drivers of follicle depletion. Top left: Ischemia–reperfusion injury induces severe oxidative stress, characterized by elevated reactive oxygen species (ROS) and mitochondrial dysfunction. Bottom left: Pathway-induced “burnout” occurs when the PI3K/PTEN/Akt and mTOR signaling pathways force dormant follicles into rapid, premature activation. (**Right**) Protective Interventions: Proposed therapeutic strategies to counteract follicle loss. Top right: A dual-drug approach combining VEGFA and Rapamycin, where VEGFA promotes tissue revascularization and rapamycin inhibits mTOR to suppress follicle over-activation. Bottom right: Targeted molecular mitigation utilizes antioxidants to reduce ROS-mediated oxidative damage, alongside mTOR inhibitors (Rapamycin) to maintain follicle dormancy. The inset table summarizes the specific biological mechanisms and primary outcomes for Antioxidants, VEGFA, and Rapamycin (Data generated using NotebookLM).

### 5.4. Scaffolds, Artificial Ovary Development, and Fibrosis Prevention

To overcome follicular depletion and malignant cell contamination risks, the field is advancing toward tissue engineering.

#### 5.4.1. Decellularized Scaffolds and Clinical Outcomes

The use of decellularized human extracellular matrix (Alloderm) scaffolds in robot-assisted ovarian tissue transplantation has demonstrated clinical success, including restoration of ovarian function, follicle development, and live births [88,89]. Oktay and colleagues reported the first pregnancies and live birth following robot-assisted transplantation using Alloderm scaffolds, with patients showing sustained ovarian function for up to 2 years post-transplantation [89]. The robotic approach may enhance precision, reduce tissue handling time, and improve graft outcomes [88].

#### 5.4.2. Biomaterial Scaffolds for Follicle Culture

Natural hydrogels and synthetic electrospun fibers do provide architectural support for follicle development. A 2026 systematic review analyzing 137 studies found that electrospun poly(ε-caprolactone) (PCL) offers superior control over architecture, reproducibility, and scalability compared to natural materials, supporting follicle survival across multiple mammalian models [90]. Natural materials like alginate and Wharton’s jelly-derived hydrogels provide tissue-specific biological cues but face limitations in mechanical stability and batch-to-batch variability [90]. Hybrid strategies combining biological activity with engineered scaffold tunability appear most promising [90].

#### 5.4.3. Decellularized Ovarian Tissue Scaffolds

Human ovarian tissue can be effectively decellularized while preserving extracellular matrix components (collagen, laminin, fibronectin), and these scaffolds support survival of isolated human pre-antral follicles both in vitro and after xenotransplantation [91]. Porcine ovarian decellularized ECM has demonstrated follicular growth, estradiol secretion, and live births in murine models, positioning it as a potential interspecies scaffold [92].

#### 5.4.4. Anti-Fibrotic Interventions

Post-transplantation fibrosis is indeed a significant challenge. Erythropoietin (EPO) administered after transplantation reduces ischemic damage, increases angiogenesis, maintains follicle proliferation, and reduces fibrotic areas in grafted ovarian tissue [82]. Pro-angiogenic factors, including VEGF and angiopoietin-2, decrease fibrosis when administered before transplantation, with combination therapy showing the greatest reduction in fibrotic area at 28 days post-grafting [93]. The TGF-β/Smad signaling pathway is central to ovarian fibrosis, orchestrating fibroblast activation and excessive ECM deposition [94]. However, clinical translation of anti-fibrotic strategies remains experimental, with most evidence derived from animal models [70].

While these technologies show promise, follicle loss remains substantial (up to 70%) due to ischemia–reperfusion injury during transplantation [84]. Standardization of decellularization protocols and rigorous translational validation remain key challenges before widespread clinical adoption [90,92].

## 6. Pregnancy After Ovarian Tissue Transplantation (OTT)

Following OTT, the recovery rate of ovarian endocrine function is remarkably high, ranging from 70% to 95%, with menstrual cycles typically resuming within 18 weeks [16]. The overall live birth rate is estimated at 26.5% to 28% [6]. Age at the time of cryopreservation is a critical prognostic factor; women under 35 achieve live birth rates of 28.2%, compared to 16.7% for those over 35. Natural conception remains the primary and most successful pathway, accounting for 66.7% of successful deliveries post-OTT [95]. When natural conception is not feasible, in vitro fertilization (IVF) serves as an essential adjunct, despite challenges such as higher empty follicle rates in grafted tissues [96]. Reassuringly, perinatal outcomes and congenital anomaly rates following OTT are comparable to the general pregnant population [97].

## 7. Conclusions

In conclusion, ovarian tissue cryopreservation (OTC) stands as the only viable fertility preservation strategy for prepubertal girls and adolescent cancer patients who cannot safely delay gonadotoxic treatments. The clinical success of this intervention relies heavily on a comprehensive pre-cryopreservation evaluation, rigorous surgical tissue harvesting, and optimized cryopreservation protocols. While slow freezing remains the clinical gold standard, vitrification demonstrates comparable efficacy and superior histological protection. Post-transplantation, orthotopic grafting facilitates robust endocrine recovery and natural conception, serving as the primary pathway to live births (Table 1).

However, the lifespan of transplanted tissue remains severely limited by ischemia–reperfusion injury and abnormal signaling activation (e.g., PI3K/PTEN/Akt, mTOR) that results in follicular burnout. Overcoming these physiological barriers necessitates targeted molecular interventions, such as the integration of antioxidants, anti-apoptotic agents, mTOR inhibitors, and pro-angiogenic factors. Looking forward, the development of multi-step in vitro maturation (IVM) and bioengineered scaffolds represents the next frontier in oncofertility. As global live birth data continues to surge, the integration of deeper molecular insights and advanced tissue engineering will be paramount in refining OTC protocols, ultimately ensuring safer and more durable fertility restoration for young cancer survivors.

## Figures and Tables

**Table 1:** Mechanisms of follicle loss and molecular strategies for fertility preservation during ovarian tissue cryopreservation (OTC) and transplantation (OTT).

Stage	Key Factors in Follicle Loss	Molecular Insights and Preventive Strategies	References
Before OTC	Age, diminished AMH levels	-	[10,11,12]
	Chemotherapy-induced damage (atresia, apoptosis, inflammation)	GnRH agonists	[40,41]
	Primordial follicle activation	mTOR inhibitors (e.g., rapamycin), AMH, S1P	[81,82,85,86,87]
Ovarian Tissue Harvest	Ischemic injury	Minimizing time to cryopreservation	[46,47]
	Primordial follicle activation	mTOR inhibitors (e.g., rapamycin), AMH, S1P	[81,82,85,86,87]
OTC	Cryodamage (slow freezing vs. vitrification)	-	[48,52,53]
	Cryoprotectant toxicity and structural damage	Biomaterials (e.g., hydrogels), anti-freeze proteins, graphene oxide (GO)	[66,67]
OTT	Suboptimal tissue preparation	Pre-treatment with estradiol, aspirin, FSH	[68]
	Site-specific microenvironment challenges	Optimization of transplantation site (e.g., retroperitoneum)	[69]
	delayed revascularization, ischemia	VEGF, erythropoietin, S1P, stem cell therapy, fingolimod	[81,82]
	Oxidative stress	Anti-apoptotic agents, antioxidants (e.g., calcium, melatonin)	[83,84,85]
	Primordial follicle activation	mTOR inhibitors (e.g., rapamycin), AMH, S1P	[81,82,85,86,87]
	Lack of structural support	Application of biocompatible scaffolds	[88]
	Stromal fibrosis	Anti-fibrotic therapies	[90,93,94]
Pregnancy after OTT	Declining ovarian reserve post-OTT	Proactive fertility management (Natural conception, IUI, IVF)	[16,95,96,97]

Abbreviations: AMH, Anti-Müllerian hormone; FSH, Follicle-stimulating hormone; GnRH, Gonadotropin-releasing hormone; GO, Graphene oxide; IUI, Intrauterine insemination; IVF, In vitro fertilization; mTOR, Mammalian target of rapamycin; OTC, Ovarian tissue cryopreservation; OTT, Ovarian tissue transplantation; S1P, Sphingosine-1-phosphate; VEGF, Vascular endothelial growth factor.

## Data Availability

No new data were created or analyzed in this study. Data sharing is not applicable to this article.
